# Isothiocyanates Are Promising Compounds against Oxidative Stress, Neuroinflammation and Cell Death that May Benefit Neurodegeneration in Parkinson’s Disease

**DOI:** 10.3390/ijms17091454

**Published:** 2016-09-01

**Authors:** Giulia Sita, Patrizia Hrelia, Andrea Tarozzi, Fabiana Morroni

**Affiliations:** 1 Department of Pharmacy and Biotechnology, Alma Mater Studiorum-University of Bologna, via Irnerio 48, 40126 Bologna, Italy; patrizia.hrelia@unibo.it (P.H.); fabiana.morroni@unibo.it (F.M.); 2 Department for Life Quality Studies, Alma Mater Studiorum-University of Bologna, Corso d’Augusto, 237, 47900 Rimini, Italy; andrea.tarozzi@unibo.it

**Keywords:** Parkinson’s disease, neuroinflammation, isothiocyanates, neuroprotection, oxidative stress, NrF2 pathway

## Abstract

Parkinson’s disease (PD) is recognized as the second most common neurodegenerative disorder and is characterized by a slow and progressive degeneration of dopaminergic neurons in the substantia nigra. Despite intensive research, the mechanisms involved in neuronal loss are not completely understood yet; however, misfolded proteins, oxidative stress, excitotoxicity and inflammation play a pivotal role in the progression of the pathology. Neuroinflammation may have a greater function in PD pathogenesis than initially believed, taking part in the cascade of events that leads to neuronal death. To date, no efficient therapy, able to arrest or slow down PD, is available. In this context, the need to find novel strategies to counteract neurodegenerative progression by influencing diseases’ pathogenesis is becoming increasingly clear. Isothiocyanates (ITCs) have already shown interesting properties in detoxification, inflammation, apoptosis and cell cycle regulation through the induction of phase I and phase II enzyme systems. Moreover, ITCs may be able to modulate several key points in oxidative and inflammatory evolution. In view of these considerations, the aim of the present review is to describe ITCs as pleiotropic compounds capable of preventing and modulating the evolution of PD.

## 1. Introduction

Neurodegenerative diseases are estimated to surpass cancer as the most important cause of death by the 2040s [1]. Neurons are the bricks that constitute the central nervous system (CNS), and they are characterized by the inability to reproduce or replace themselves. In this way, when damage threatens, the loss of cells will be irreversible. The term neurodegeneration defines a variety of conditions that modify normal neuronal functions in the human brain, where it is possible to observe a progressive and consistent cells loss. This definition identifies different pathologies, such as Alzheimer’s disease (AD), Parkinson’s disease (PD), Huntington’s disease (HD), amyotrophic lateral sclerosis (ALS), and so forth. The mechanisms involved in the evolution of these conditions are not completely understood yet; however, they share common characteristics, such as misfolded proteins, oxidative stress, inflammation, excitotoxicity and, obviously, neuronal loss [2,3]. These processes can occur both in sporadic and familial cases, which highlights how these pathological mechanisms are strongly correlated with neuron dysfunctions [4]. Patients are usually affected later in life; for this reason, in the last 20 years, the number of diagnoses is prominently enhanced, especially for the increase in life expectancy. However, numerous evidences suggest the presence of a preclinical asymptomatic stage that could begin years before the establishment of the first symptoms. This pre-symptomatic stage could be considered the key phase, because it represents the time point where it is possible to modulate the development of the disease [5]. Unfortunately, the current therapy is only symptomatic, and it could not arrest the progression of the neurodegenerative process [6].

Inflammatory processes have been implicated in neurodegenerative conditions [7]. Inflammation is a double-faced mechanism: on the one hand, secretory factors, such as cytokines and chemokines, cause an increased cerebral blood flow to the affected area and the removal of damaged tissue by phagocytic cells; on the other hand, they increase the production of reactive oxygen species (ROS) and lead to the destruction of the blood brain barrier (BBB) [8].

Isothiocyanates (ITCs) from cruciferous vegetables have already shown interesting properties in the induction of phase I and II enzymes involved in detoxification processes, and they have demonstrated the ability to interfere in the cell cycle regulation promoting the apoptotic pathway [9,10]. Furthermore ITCs are able to modulate the oxidative condition and the inflammatory process [11,12]. These compounds have been widely studied as antitumoral agents [13], and they have also shown interesting effects against cardiovascular [14,15,16], CNS [17,18] and skin diseases [19].

In light of these considerations, the aim of the present review is to describe ITCs as potential neuroprotective compounds in the prevention of PD. In particular, we have summarized the effects of sulforaphane (SFN), erucin (ER), phenethyl isothiocyanate (PEITC) and 6-(methylsulfinyl)hexyl isothiocyanate (6-MSITC) on the evolution of PD.

## 2. Parkinson’s Disease

In 1817, James Parkinson described, in his monography “Essay in the Shaking Palsy”, the essence of the second most common age-related neurodegenerative disorder after AD [2]. PD is a neurodegenerative multisystem disorder afflicting approximately six million people worldwide and characterized by a combination of motor and non-motor symptoms. The main pathological modifications in the parkinsonian brain are the degeneration of dopaminergic neurons of the substantia nigra pars compacta (SNpc) in the ventral midbrain and the depletion of dopamine (DA) in the striatum (STR). SNpc and STR are part of a complex neuronal system that includes cortex, basal ganglia and thalamus, and it is responsible for the normal motor functions. While the neurons of this region are preferentially lost in PD, they are not the only ones involved in the progression of the disease. In addition to neuronal loss, the condition is associated with an accumulation of cytoplasmic, eosinophilic neuronal inclusions, known as Lewy bodies (LBs), predominantly composed of the presynaptic protein α-synuclein (α-syn) [2,20]. The exact biological function of this protein and the mechanism by which it leads to neuronal loss are still not clear, although it has been observed that an excess of α-syn depositions can cause dopaminergic neuronal death [21].

PD is characterized by cardinal motor symptoms, including bradykinesia, rigidity, postural instability and resting tremor. During early stages of PD, patients also exhibit non-motor manifestations, such as autonomic dysfunctions, cognitive abnormalities, sleep and mood disorders, pain and sensory conditions reflecting the peripheral neurodegeneration [22]. Despite the increasing evidence of their importance on the quality of life, studies have shown that there are several difficulties in the treatment of these issues [23].

The current therapy for PD is the administration of 3,4-dihydroxyphenylalanine (L-DOPA), a precursor of DA. The pathology evolution determines that dopaminergic neurons are continuously diminished, and the treatment loses efficacy also because it cannot find its own target. Anyway, the therapy is only palliative and symptomatic, as it cannot arrest the progression of the disease, but it can only supply the lack of DA [24]. However, there are many symptoms that do not respond to the dopaminergic therapy. Moreover, the transformation of L-DOPA to DA may contribute to the increment of ROS and consequently to the progression of the neurodegenerative process.

### Mechanisms Involved in Parkinson’s Disease

Oxidative stress is defined by the imbalance between the levels of ROS produced and the capacity of biological systems to eliminate or to quench the reactive intermediates formed. Within evolution, all biological systems have developed adaptive responses that include defensive enzymes, molecular chaperones and antioxidant molecules [25]. Several pathway are responsible for ROS formation, such as indirect pathways involving the activation of enzymes, like nitric oxide synthase (NOS) or nicotinamide adenine dinucleotide phosphate (NADPH) oxidase, or by direct interactions between oxygen species and redox active metals [26]. The brain is the organ that consumes the largest percentage of oxygen introduced into the body, with an amount close to the 20%. From the moment that a significant portion of that oxygen is converted into ROS, this extensive production could provide an explanation for the crucial role of ROS in PD onset and evolution. On the other hand, the CNS is characterized by low levels of glutathione (GSH), the most important endogen antioxidant system, and by high levels of iron and calcium in the SNpc. This condition is essential to establish the redox imbalance [27,28]. Additionally, polyunsaturated fatty acids are present in high concentrations in the brain, resulting in the generation of toxic products and lipid peroxidation.

Another important mechanism involved in the pathogenesis of PD is excitotoxicity, which plays a pathological role that critically contributes to the exacerbation of nigrostriatal degeneration [29]. In PD, the altered neurotransmission observed within the basal ganglia affects the glutamatergic system, suggesting a critical involvement of glutamate-mediated excitotoxicity in the pathogenesis. Accumulation of extracellular glutamate and increased stimulation of glutamatergic neurons spreads ROS production. The activation of glutamate receptors involves calcium homeostasis dysfunction, caspases activation and determines an increase in cytotoxic transcription factors and free radical species [30].

Apoptosis is considered as the dominant mechanism of cell death in PD [31]. This process is characterized by morphological and biochemical alterations involving nuclear chromatin, cytoplasmatic organelles and endoplasmic reticulum [32]. The apoptotic process is characterized by two main pathways, extrinsic and intrinsic, that are constituted by a different set of initiator caspases, but they have the same executioners. Autophagy is another cell death mechanism involved in PD. Chaperone-mediated autophagy (CMA), which degrades only soluble forms of protein, is responsible for the α-syn recycle. A percentage of intracellular α-syn modifies the CMA pathway. Instead of entering for degradation, they accumulate in the cytoplasm, causing cell death [4]. On the other hand, necrotic cell death does not play a crucial role in a slowly progressive neurodegenerative disorder, such as PD. Interestingly, in many PD animal models, necrosis is induced by toxins when given in high doses or for long treatments. Thus, it is possible to underlie how the experimental conditions are determinant to produce apoptosis or necrosis [33].

## 3. Neuroinflammation in the Pathogenesis of PD

The term “inflammation” defines a biological response by the immune system to an insult that is physical, toxic or infective [34]. The CNS differs in its inflammatory response to the other tissues. In general terms, cellular infiltration in the brain in response to inflammation is weak and delayed; however, many inflammatory responses can be induced rapidly [35]. These include the activation of microglia and the expression and release of classic inflammatory mediators, such as acute phase proteins, eicosanoids, proteins of the complement and cytokines [36]. Chronic inflammation is a contributing feature of many neurodegenerative diseases [37]. In fact, different markers have been observed in the SNpc and STR of PD patients [38]. In 1988, McGeer and colleagues provided the first link between inflammation and PD, showing an increase of human leucocyte antigen D related (DR)-positive microglia in the substantia nigra (SN) of patients [39]. In recent years, it has been demonstrated that there is an increase in proinflammatory mediators’ activation also in the STR [40]. Although it has been studied for years, the inflammatory response related to PD has not been clarified yet, especially from the molecular angle. It is now known that PD is characterized by the accumulation of microglia and astrocytes and T cell infiltration associated with modifications in glial cell morphology and function. Considering the involvement of neuroinflammation in the progression of PD, several studies showed the potential use of anti-inflammatory drugs to reduce the risk of an individual developing PD [41,42]. Unfortunately, these studies have generated conflicting data [43], and preclinical studies on neuroinflammatory processes have not been translated into clinical practice yet.

### 3.1. Microglia as a Mediator of Neuroinflammation in PD

Different populations of macrophages are present in healthy brain tissue, each with a distinct phenotype and morphology. The most abundant of these macrophages are microglia, the resident immune cells of the brain parenchyma [44]. In the healthy brain, microglia are in a resting state characterized by a ramified morphology that permits receiving signs of distress from the tissue environment [45]. Following a brain insult, the tight junctions between endothelial cells become permeable, allowing peripheral immune cells to infiltrate brain parenchyma [46]. At the same time, immunogenic molecules are released, leading microglia to adopt an activated morphology and to secrete proinflammatory factors, including interleukin (IL)-1β, tumor necrosis factor (TNF)-α and ROS [45]. The normal activation protects neurons from injuries and maintains homeostasis in the brain. After the resolution of the activation, microglia return to the resting state.

Although the link between microglia activation and dopaminergic neuron death in PD is still under investigation, PD patients and animal models have shown that persistent microglia activation causes deleterious effects, which lead to the loss of dopaminergic neurons [47,48].

One of the main damaging effects mediated by microglia is the activation of the nuclear factor-κB (NFκB) pathway and the consequently increased release of proinflammatory cytokines, such as IL-1β and TNF-α [49,50]. Among the cytotoxic mechanisms induced by proinflammatory cytokines in PD, the activation of inducible nitric oxide synthase (iNOS) is the best clarified. iNOS produces high levels of NO and superoxide radicals, which can directly or indirectly induce neuronal death in PD brain [51]. Thus, the study of the plasticity of microglia response, which could be deleterious or beneficial for brain health, may be a useful tool to interfere with PD progression.

### 3.2. Astrocytes as Mediator of Neuroinflammation in PD

Astrocyte reaction is another well-documented neuropathological characteristic in the SNpc of PD patients [43,44]. Astrocytes form the glia around blood vessels, limiting the entry of immune cells into the CNS through BBB [52]. Interesting evidence showed the emerging role of astrocytes in neuroinflammation associated with PD [53]. As shown by Wu et al. in 2002, the SNpc of postmortem PD patients presented an increased number of astrocytes and glial fibrillary acidic protein (GFAP immunoreactivity [54]. This study demonstrated that the amount of GFAP-positive astrocytes is inversely proportional to the number of dead dopaminergic cells, so neurons are more susceptible to the neurodegenerative process if there is a lack of astrocytes [55]. It has been also discussed that the decreased integrity of astroglial cells, possibly related to the accumulation of nigral α-syn, could actually contribute to the loss of nigral neurons [56]. Microglia and astrocytes are both involved in the neuroinflammatory response, and usually, they are converted to the reactive isoform in concert; for this reason, it is not easy to distinguish their contribution to neuroinflammation. Astrocytes activation is considered as a response that although initially beneficial, is later corrupted by disease alterations [57]. From the moment that neuronal damage can be activated or be prevented by astrocytes, the imbalance between these actions could be crucial for the onset and progression of PD [53,56]. The comprehension of the role of astrocytes in PD may be essential to improve therapies and to develop new drugs for PD treatment.

### 3.3. T Cell Infiltration as a Mediator of Neuroinflammation in PD

The protection exerted by the BBB makes the brain an immune-privileged system. However, the peripheral leukocyte migration and infiltration occur in PD, probably because both the animal model and clinical syndrome exhibit BBB dysfunction. Different studies have demonstrated the infiltration of immune cells in the brain, which consisted of a heterogeneous population of both CD8+ and CD4+ cells [58]. Compelling evidence suggested that this infiltration of peripheral lymphocytes through BBB occurred because activated microglia in PD brains release proinflammatory cytokines that act on the BBB and attract lymphocytes [59]. In addition, there are studies that indicate that nitrated α-syn activated peripheral leukocytes, stimulating them to expand into different T cells, and mediated adaptive immune responses in potentiating microglial activation and exacerbating neuronal death [60]. Recently, Brochard et al. demonstrated that the removal of CD4+, but not of CD8+ T cells, in mice greatly reduced 1-methyl-4-phenyl-1,2,3,6-tetrahydropyridine (MPTP)-induced nigrostriatal cell death. These studies highlighted that the adaptive immune system, like the innate immune system, may actively participate in the pathogenesis of PD. However, more work needs to be done to find out how they will be a potential target for PD therapy.

Taking into account the multifactorial nature of PD pathogenesis, positive effects from anti-inflammatory drugs could only be possible if their administration were combined with compounds targeting other deleterious pathways, such as anti-apoptotic or antioxidants, or it would be even more exciting to find a pleiotropic compound that embodies all of these benefits.

## 4. Isothiocyanates and PD

A neuroprotective strategy proposes natural antioxidant molecules as an alternative form of treatment for the prevention of neurological pathologies, such as PD [61]. In this context, vegetables from the cruciferous family have generated interest as having potential neuroprotective compounds. The *Brassica* family is the largest and most widely-consumed group of plants in Europe and all over the world. This family includes around 340 genera and about 3700 species characterized by different levels of nutrients [62]. The beneficial effects of *Brassica* vegetables on human health have been linked to phytochemicals that prevent oxidative stress, induce enzymes of detoxification, stimulate the immune system, decrease the risk of cancer and reduce the proliferation of cancer cells, as well [63].

*Brassica* vegetables contain many valuable metabolites and are a considerable source of antioxidants. The attention reserved for these vegetables is due to their unique constituents, glucosinolates. They present three different components: a β-thioglucose part, a sulfonated oxime and a variable aglycone side chain derived from α-amino acid [64]. Glucosinolates are not bioactive compounds, and in normal conditions, they are hydrolyzed slightly. When plant tissue damage occurs by disruption, glucosinolates are hydrolyzed quickly upon a β-thioglucoside glucohydrolase enzyme, called myrosinase. This enzyme is located in myrosin cells, and the resulting product is a highly unstable aglycone intermediate that leads to the production of thiocyanates, nitriles, goitrin, epithionitriles and ITCs [65]. The spatial separation of myrosinases from glucosinolates is the basis of the glucosinolate-myrosinase system that has been described as the plants’ protective factor from insect and pathogen insults [66]. Given that myrosinases are sensitive to mild heating and cooking, when humans consume cruciferous vegetables, the main source of myrosinase activity derives from gut microflora [67]. ITCs are reactive electrophiles that are able to modify proteins that are crucial to activate noxious responses, interfere with carcinogen metabolism, reduce tumor development and modify the production of inflammatory cytokine [13,49]. Accumulating evidence has suggested that their activities are expressed through signaling pathways involved in detoxification, inflammation, apoptosis and cell cycle regulation by the induction of enzyme systems of phases I and II [68]. Indeed, the central electrophilic carbon of ITCs (R–N=C=S) undergoes rapid addition reactions with biological nucleophiles. Drobnica et al. demonstrated the ability of ITCs to react with thiol groups up to one thousand times faster than with amino groups. This ability made proteins with cysteine residues particularly sensitive targets for modification by ITCs [69]. ITCs are initially conjugated to GSH through catalysis by glutathione-*S*-transferase (GST). Once ITCs are conjugated, they are rapidly effused from cells, and the acetylation that follows the removal of glutamate and glycine generates a mercapturic acid (*N*-acetylcysteine isothiocyanate) residue excreted into urine. However, in all of these compounds, the most important characteristic is that the cysteine-isothiocyanate conjugate is able to dissociate back to the original ITCs [70]. This reversibility is fundamental to transport them throughout the body and to enhance the reaction of free ITCs with more reactive targets [71]. In cells, the protective responses to oxidative damage are essentially three: the cis-acting enhancer sequence termed the antioxidant response element (ARE) that is included in genes, such as heme oxygenase-1 (HO-1), the transcription factor nuclear factor (erythroid-derived 2)-like 2 (NrF2) that is considered as a guardian of the cellular redox homeostasis and the cytosolic NrF2-repressor protein, the Kelch enoyl-CoA hydratase (ECH)-associated protein 1 (Keap1), characterized by the high content of cysteine groups [11,71,72]. Under normal conditions, NrF2 is held by Keap1 in the cytoplasmatic fraction. In the presence of an oxidative stimulus, NrF2 is released and translocated into the nucleus, where it heterodimerizes with the small Maf proteins enhancing the binding to ARE genes in the promoter regions. This event induces the NrF2/ARE pathway at the transcriptional level to restore homeostasis through upregulation of phase II detoxification enzymes [11,68,72,73].

Several in vitro studies clearly indicated that the activation of NrF2 results in the protection against hydrogen peroxide (H_2_O_2_)-induced oxidative stress [74]. As shown by Ramos-Gomez et al., the critical role of NrF2 is highlighted by its absence, in this case, the sensitivity to xenobiotics spreads and oxidative stress is increased, leading to the amplification of cytokine production, as NF-κB is more readily activated in oxidative environments [75,76]. Animal models of PD showed a dramatic loss of dopaminergic neurons in transgenic animals with NrF2 knockout, while the overproduction of this enzyme or the downregulation of Keap1 have neuroprotective effects on them [77,78]. A similar increase in the sensitivity of the NrF2-deficient mice has been observed in the 6-hydroxydopamine (6-OHDA) model both in vitro and in vivo; in particular, there was a more significant neurodegeneration in the presence of NrF2 deficiency than in wild-type counterparts [79]. Similarly, also in the 1-methyl-4-phenyl-1,2,3,6-tetrahydropyridine (MPTP) animal model, researchers observed a more impactful loss of DA in the STR of NrF2 knockout mice than in the wild-type at different MPTP doses [80,81]. It is well known that the NrF2 and NFκB pathways could interplay through multiple molecular interaction, and their imbalance is associated with different diseases, ranging from cancer, autoimmune disorders and neurodegeneration [82]. The modulation of NrF2 in response to NFκB activation could be a protective mechanism against the consequences of inflammation [83]. There are several mechanisms by which p65 (the canonical NFκB subunit) can exert a negative role in the regulation of NrF2 activity. For instance, Rushwort et al. [84] demonstrated that NrF2 contains several κB sites in its promoter, which are regulated by p65. In addition, p65 can induce the repression of transcription by deacetylation of histones, associating with histone deacetylase [85]. Finally, Jiang et al. [86] showed in 2014 that the increase in Nrf2 activity prevents the activation of p65, by accumulation of GSH [86]. However, despite the known evidence for functional interactions between these two pathways, many aspects of the nature of their cross-talk require further investigations.

**Figure 1:**
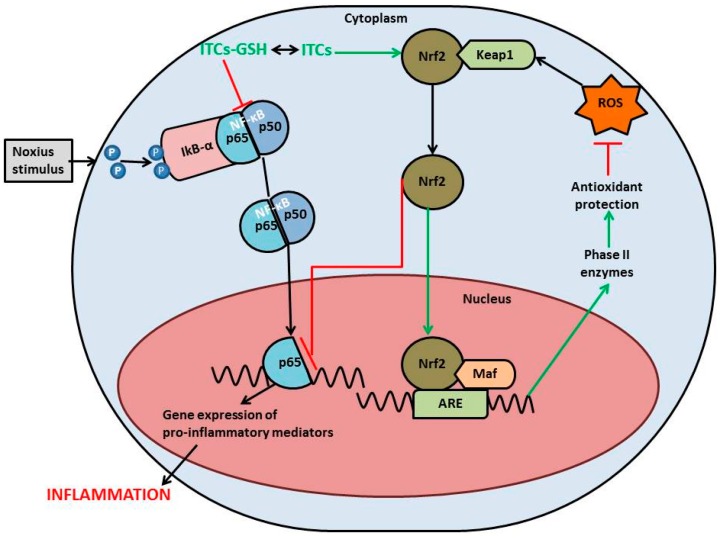
Summary of the main mechanisms involved in the neuroprotective activity of isothiocyanates (ITCs). ITCs are known not only to be potent NrF2 activators and to exhibit antioxidant effects via the upregulation of ARE-driven genes, but also to decrease the inflammatory response through the NFκB pathway. Red arrows: blocked pathways; green arrows: enhanced pathways; black arrows: normal pathway of NFκB.

The relevance of the NrF2/HO-1 axis in downregulation of brain inflammation and the design of drugs that could pass through the BBB to modulate NrF2 activity are fundamental issues that need to be solved [87]. ITCs are known not only to be potent NrF2 activators and to exhibit antioxidant effects via the upregulation of ARE-driven genes, but also to decrease the inflammatory response through the NFκB pathway [69]. In oxidative conditions, NFκB is activated, resulting in further expression of pro-inflammatory cytokines [88]. Once NFκB is released, it is accumulated in the nucleus, where it induces the transcription of target genes involved in cell inflammatory responses [89]. The suggested neuroprotective mechanism of ITCs is summarized in Figure 1. Even if the interaction between ITCs and Keap1 is still under investigation, it might be triggered by protein kinases or as a direct reaction of ITCs with the sulfhydryls groups of cysteine residues in Keap1, both mechanisms result in conformational changes responsible for the release of NrF2 that can move to the nucleus [90].

ITCs and SFN in particular have demonstrated anti-inflammatory activity, as shown by Heiss et al. in the RAW 264.7 macrophage cell line [12]. The authors showed that SFN was able to inhibit NFκB release and the transactivation of κB-dependent genes. The capacity to downregulate the NFκB pathway during inflammation was demonstrated also by Brandenburg et al. in a study aimed to investigate the capacity of SFN to inhibit the signal transduction pathways involved in response to LPS insult on primary rat microglia and on murine microglial cells, recombinant retrovirus (v-raf/v-mic) transformed (BV2) [91].

### 4.1. Sulforaphane

SFN, one of the most studied ITCs, is a natural compound highly present in cruciferous vegetables, like broccoli, cauliflower, Brussel sprouts and cabbage. Interestingly, it has shown protective effects on neurons, decreasing both microglia activation and the upregulation of inflammatory markers following the endotoxins’ injection [87,92,93,94]. Jazwa et al. showed in an MPTP mouse model of PD that the intraperitoneal (i.p.) injection of SFN led to an upregulation of phase II antioxidant enzymes (HO-1, NQO1) and to an increase of NrF2 proteins’ level in basal ganglia [95]. In addition, the intrathecal (i.t.) administration of SFN reduced astrogliosis and the expression of cytokines after spinal nerve transection; moreover, it counteracted the development of neuropathic pain [96]. The anti-inflammatory properties of SFN has been well-documented in studies conducted on NrF2^−/−^ macrophages treated with LPS, in which there was a consistent increase in proinflammatory markers, such as cyclooxygenase-2 (COX-2), iNOS, IL-6 and IL-8 [97,98]. At the same time, SFN was able to reduce astrocytes and microglia activation, as shown by Jazwa et al. [95]. In our laboratory, Tarozzi et al. demonstrated that the elevation of GSH in SH-SY5Y cells treated with 6-OHDA after a pretreatment with SFN could be determinant to prevent the early redox status impairment responsible for mitochondrial dysfunction and apoptosis [99]. We have also demonstrated that the i.p. administration of SFN in a 6-OHDA murine model of PD was able to increase GSH and related enzymes levels, as well as to modulate extracellular signal-regulated protein kinase 1/2 (ERK1/2) activity [18]. Moreover, Deng et al. showed the capacity of SFN to counteract toxicity induced by 6-OHDA through the activation of the NrF2-ARE pathway [100,101]. Thus, the neuroprotective activity of SFN is associated with a mechanism involving nuclear translocation of NrF2 and the subsequent increase of phase I and II detoxifying enzymes, with simultaneously activation of ERK 1/2 and Akt pathways [102,103], and finally, with the ability to counteract neuroinflammation [104].

Therefore, due to the relation between NrF2/ARE pathway and SFN mechanism of action, we can affirm that it could be an excellent candidate for the modulation of brain inflammation and oxidative stress in neurodegenerative conditions, such as PD.

### 4.2. Erucin

Although much research has focused mainly on SFN, other ITCs, such as erucin (ER), are promising. ER (4-(methylthio)butyl ITC) is a hydrolysis product of glucoerucin (GER), which is found in arugula, and an in vivo reduction product of SFN. Clarke et al. showed the interconversion of SFN into ER in the urine of individuals consuming fresh broccoli sprouts or supplements [105]. Although the biological effects of ER are still poorly investigated, this compound has shown promising efficacy for anticancer in several studies [106,107]. Different mechanisms of chemoprevention, such as the alteration of phase I, II and III enzymes, regulation of cell proliferation and downregulation of androgen receptor signaling, can be exerted by ER [108]. In a recent study conducted by Yehuda et al., also the anti-inflammatory effects of ER were investigated. The study reported the ability of this ITC to decrease the transcription of proinflammatory molecules (TNF-α, IL-1β and IL-12) in THP-1 human acute monocytic leukemia cells, treated with LPS [109]. In addition, Cho et al. demonstrated in LPS-stimulated macrophages its potent anti-inflammatory properties mediated through the inhibition of NFκB signaling. They also showed the ability of ER to inhibit inflammatory responses efficiently in mouse skin at low doses. These results suggest the potential of this ITC as an anti-inflammatory agent [110]. In addition, Wagner et al. showed the effect of myrosinase-treated glucoerucin (GER), which releases ER, on HO-1 expression and NrF2 signaling in cultured cells and in mice. They suggested that ER induced NrF2 via ERK1/2-, p38- and JNK-dependent signal transduction pathways and HO-1 expression primarily through p38 signaling [111]. In our previous study, we demonstrated that ER prevented the oxidative damage induced by 6-OHDA in SH-SY5Y cell line. At the cytosolic level, ER increased both GSH and the antioxidant capacity. The increased resistance to apoptotic neuronal death in SH-SY5Y cells has been linked to higher levels of GSH [112]. ER showed an interesting profile of antioxidant, anti-inflammatory and neuroprotective effects similar to those observed with SFN. These findings suggested that the neuroprotective effects of SFN recorded in various animal models could also be ascribed to its metabolic conversion to ER. Due to the lack of data on animal models of neurodegeneration, further studies are warranted to investigate the potential in vivo neuroprotective role of ER.

### 4.3. Phenethyl Isothiocyanate

Phenetyl isothiocyanate (PEITC) is an organosulfur bioactive compound that has been found in cruciferous vegetable, especially in seeds of winter cress. PEITC is released from its glucosinolate precursor gluconasturtiin (GNS) by enzymatic hydrolysis [113]. This ITC has attracted researchers’ attention because of its high potency against several tumors, such as those of the lung, breast, pancreas and colon [114,115]. In addition, PEITC presents a low in vivo toxicity, as shown in animal models treated with equivalent anticarcinogenic doses or at higher doses [116]. PEITC is characterized by a high oral bioavailability and fast absorption probably due to the low molecular weight [114]. PEITC has shown potent antioxidant and anti-inflammatory activities [117]. In addition, PEITC showed a low clearance rate. After absorption, the molecule is rapidly metabolized mainly through the mercapturic acid pathway [118]. PEITC reacts with GSH, forming the phenethyl isothiocyanate-glutathione (PEITC-GSH) conjugate catalyzed by GST in the cytosol [119]. Several studies reported the ability of PEITC to modulate inflammation by the suppression of the expression of iNOS, COX-2 and IL-1β macrophages [117,120]. In 2013, Park et al. demonstrated that PEITC suppressed the target gene expression, IP-10, induced by lipopolysaccharide (LPS) and the transcriptional activation of interferon regulatory factors 3 (IRF3) by inhibiting the interferon-β (TRIF)-dependent signaling pathway of Toll-like receptors [121]. The anti-inflammatory action of this ITC is also related to the ability to interact with the NrF2 pathway, as shown by Boyanapalli et al. [122]. They used a transgenic mouse knockout model for NrF2 to isolate peritoneal macrophages that were pretreated, after isolation, with PEITC and lesioned with LPS. The results obtained showed that the induction of NrF2 is essential to obtain a response by PEITC treatment in inflammation countering [122].

### 4.4. 6-(Methylsulfinyl)hexyl Isothiocyanate

Wasabi (*Wasabia japonica*) is a member of the Brassicaceae family of vegetables, and its rhizome is a very popular pungent spice in Japan. Wasabi differs from other Brassicaceae species because it contains a higher concentration of long-chain ITCs. The major compound is the 6-(methylsulfinyl)hexyl isothiocyanate (6-MSITC) that has shown the ability to inhibit proinflammatory and prooxidant factors [17,123]. Several studies demonstrated that COX-2 and NO activations by LPS are efficiently counteracted by 6-MSITC treatment in a murine macrophage cell line [124,125]. Chen et al. showed that 6-MSITC modulated several genes associated with inflammatory responses, regulating also cytokines expression [126]. In animal experiments, 6-MSITC is rapidly absorbed into the body and induces hepatic phase II detoxification enzymes more potently than SFN [127]. In the same study, the authors showed that 6-MSITC is a potential activator of the NrF2/ARE-dependent detoxification pathway, by activating the antioxidant response element (ARE) and inducing nuclear localization of NrF2. Moreover, Mizuno and colleagues investigated the effect of SFN and 6-MSITC on oxidative stress-induced cytotoxicity using primary neuronal cultures of rat striatum [128]. They demonstrated that 6-MSITC was more potent than SFN in inducing the expression of γ-glutamylcysteine synthetase and HO-1. However, they could find no difference for the cytoprotective effect and for the modulation of intracellular GSH between SFN and 6-MSITC. Results obtained in our laboratories highlighted that the administration of 6-MSITC for one month exerted neuroprotective effects in the 6-OHDA model of PD. Indeed, 6-MSITC treatment resulted in a significant decrease in oxidative stress and apoptotic cell death, leading to an improvement of behavioral impairments [17]. These findings suggested that this ITC may be a promising neuroprotective compound against neurodegeneration that occurs in PD.

### 4.5. Novel Compounds

Naturally-occurring ITCs are known to possess chemopreventive and neuroprotective properties. Recent studies investigated the effects of synthetic ITCs in order to ameliorate SFN safety, in the case that SFN cytotoxicity will become a concern in future therapeutic applications. Researchers tried to optimize natural compound efficacy by substitution of the aromatic rings in place of the methyl group on the SFN structure. A good example is the novel compound ITC-3 synthetized by Lee et al. that led to the elevation of nuclear and total levels of the transcription factor NrF2 and interacted with its binding protein Keap1 with high affinity. At the same time, ITC-3 was able to reduce the production of the proinflammatory mediators significantly in LPS-activated BV-2 microglial cells [129]. Finally, in the same study, the authors showed its neuroprotective activity in the MPTP mouse model of PD, by attenuating the loss of tyrosine hydroxylase-immunopositive nigrostriatal DAergic neurons, suppressing microglial activation and abolishing PD-associated motor deficits.

Another interesting synthetic ITC is 4-iodophenyl isothiocyanate (4-IPITC). Wellejus et al. showed its neuroprotective activity on primary cortical neurons exposed to various insults, such as excessive glutamate exposure, oxygen-glucose deprivation, oxidative stress and 1-methyl-phenylpyridinium (MPP^+^). Moreover, they studied the effects of 4-IPITC in a murine PD model. Although the number of animals in this MPTP study was limited, 4-IPITC showed the potential to counteract neurotoxicity [130]. Taking into account these considerations, novel compounds that target both oxidative damage and inflammatory response would be useful toward the development of a disease-modifying therapy for PD.

## 5. Conclusions

Neuroinflammation is, together with oxidative stress, one of the most preponderant contributors to neurodegenerative diseases. The tight correlation between these factors is responsible of the vicious cycle that is easily observed in the evolution of neurodegenerative diseases. In diseases such as PD, where the multifactorial nature is now recognized, the importance to find out an efficient multitarget therapy is determinant to counteract the diseases’ evolution. To date, no efficient therapy, able to arrest or slow down PD, is available. In this view, ITCs have shown an interesting ability to modulate oxidative stress and inflammatory processes (see Table 1 and Table 2 for a summary). Due to the capacity of these compounds to interact with the NrF2 pathway, new studies are projected to evaluate new molecules synthetized from the ITCs’ structure to improve chemical characteristic, with the aim to find drugs able to counteract the neurodegenerative processes’ evolution efficiently.

## Figures and Tables

**Table 1:** Summary of experimental models in vitro and isothiocyanate (ITC) doses.

Model	Damage		Isothiocyanates		References
	Toxins	Doses	Molecules and Sources	Doses	
Primary peritoneal macrophage from NrF2^−/−^ mice	LPS ^1^	1 µg/mL	SFN ^2^	5–40 µM	[97]
SH-SY5Y cell ^3^	H_2_O_2_^4^	300 µmol/L	SFN	0.63–5 µmol/L	[99]
	6-OHDA ^5^	100 µmol/L			
PC12 ^6^ cell line	6-OHDA	80 µM	SFN	5 µM	[100]
Primary cortical neurons	5-*S*-cysteinyl-dopamine	100 µM	SFN	100 nM	[102]
CATH.a cell ^8^	BH_4_^7^	200 µM	SFN	0.5–5 µM	[93]
	6-OHDA	50 µM			
	MPP^+^^9^	2 mM			
	AMPT ^10^	100 µM			
SK-N-BE(2)C cell ^11^	BH_4_	200 µM			
	6-OHDA	50 µM			
	MPP^+^	2 mM			
	AMPT	100 µM			
RAW 264.7 ^12^	LPS	1 mg/L	ER ^13^	2.5–5 µmol/L	[110]
HT-29 cell ^14^			GER ^15^	25 µmol/L	[111]
			GRA ^16^		
			SIN ^17^		
			GST ^18^		
SH-SY5Y cell	6-OHDA	200 µmol/L	ER	1.25–5 µmol/L	[112]
Human plasma and urine			Watercress	100 g	[114]
RAW 264.7	LPS	1 µg/mL	PEITC ^19^	100 mM	[117]
			MSO ^20^		
RAW 264.7	LPS	500 ng/mL	SFN	0.4–50 µM	[12]
RAW 264.7	LPS	1 µg/mL	*Barbarea verna*	1–20 µM	[120]
HL60 cell line ^21^			PEITC metabolites	0.1–100 µM	[118]
RAW 264.7	LPS	110 ng/mL	PEITC	20 µM	[121]
Peritoneal macrophages of C57BL/6 mice	LPS	1 µg/mL	PEITC	5–10 µM	[122]
Peritoneal macrophages of NrF2^−/−^ mice					
Primary cortical neurons from E18 Wistar rats	Glutamate	20 µM	4-IPITC ^22^	0.1–10 µM	[130]
	H_2_O_2_	45 µM			
Mesencephalon neurons	MPP^+^	4 µM			
RAW 264.7	LPS	40 ng/mL	6-MITC ^23^	16 µM	[124]
RAW 264.7	LPS	40 ng/mL	6-MITC	8 µM	[126]
RL34 cells ^24^			6-MITC	25 µmol	[127]
Primary neurons of rat striatum	Paraquat	200 µM	SFN	0.01–1 µM	[128]
	H_2_O_2_	30 µM			
	Paraquat	200 µM	6-MITC		
	H_2_O_2_	30 µM			
CATH.a cell	MPP^+^		ITC-3	0.05–1 µM	[129]
	BH_4_				
BV-2 cells	LPS	0.2 µg/mL		1–20 µM	

Abbreviations: ^1^ Lipopolysaccharide; ^2^ sulforaphane; ^3^ neuroblastoma cell line; ^4^ hydrogen peroxide; ^5^ 6-hydroxydopamine; ^6^ pheochromocytoma rat adrenal medulla derived cell line; ^7^ tetrahydrobiopterin; ^8^ catecholaminergic cell line; ^9^ 1-methyl-4-phenylpyridinium; ^10^ α-methyl-p-tyrosine; ^11^ neuroblastoma cell line; ^12^ mouse leukemic monocyte macrophage cell line; ^13^ erucin; ^14^ human Caucasian colon adenocarcinoma grade II cell line; ^15^ glucoerucin; ^16^ glucoraphanin; ^17^ sinigrin; ^18^ gluconasturtiin; ^19^ phenethyl isothiocyanate; ^20^ 8-methylsulfinyloctyl; ^21^ human promyelocytic leukemia cells; ^22^ 4-iodophenyl isothiocyanate; ^23^ 6-(methylsulfinyl)hexyl isothiocyanate; ^24^ rat liver epithelial cell line.

**Table 2:** Summary of experimental models in vivo and isothiocyanate (ITC) doses.

Model	Damage		Isothiocyanates		References
	Toxins	Doses	Molecules and Sources	Doses	
NrF2^−/−^ mice	MPTP ^1^	30 mg/kg	SFN ^2^	50 mg/kg	[95]
Nox2^−/−^ mice	Spinal nerve transection		SFN	10 mg/kg	[96]
				50 mg/kg	
CX_3_CR_1_^3+/GFP^ mice				10 mg/kg	
				50 mg/kg	
NrF2^−/−^ mice			Broccoli seeds	15% on weight	[92]
C57BL/6 mice	6-OHDA ^4^	2 µL, 4 µg/mL	SFN	5 mg/kg	[18]
Sprague-Dawley rats	OKA ^5^	200 ng	SFN	5 mg/kg	[104]
ICR mice	TPA ^6^	5 nmol	ER ^7^	100–300 nmol	[110]
C57BL/6 mice			GER ^8^	20 mg/kg	[111]
			GRA ^9^		
			SIN ^10^		
			GST ^11^		
Wistar rats	λ-Carrageenan	100 µL, 1%	PEITC ^12^ oil	200 mg/kg	[120]
Dark agouti rats	Myelin oligodendrocyte glycoprotein	0.7 mg/mL		10–40 mg/kg	[130]
C57BL/6 mice	MPTP	20 mg/kg		5 mg/kg	
C57BL/6 mice	MPTP	40 mg/kg	RS-GRA	10 mg/kg	[94]
		20 mg/kg			
ICR mice			SFN	25 µmol	[127]
			6-MITC ^13^		
NrF2^−/−^ mice					
C57BL/6 mice	6-OHDA	2 µL, 4 µg/mL	6-MITC	5 mg/kg	[17]
C57BL/6 mice	MPTP	20 mg/kg	ITC-3	30 mg/kg	[129]

Abbreviations: ^1^ 1-Methyl-4-phenyl-1,2,3,6-tetrahydropyridine; ^2^ sulforaphane; ^3^ fractalkine receptor; ^4^ 6-hydroxydopamine; ^5^ okadaic acid; ^6^ 12-*O*-tetradecanoylphorbol-13-acetate; ^7^ erucin; ^8^ glucoerucin; ^9^ glucoraphanin; ^10^ sinigrin; ^11^ gluconasturtiin; ^12^ phenethyl isothiocyanate; ^13^ 6-(methylsulfinyl)hexyl isothiocyanate.
